# The Cellular Thioredoxin-1/Thioredoxin Reductase-1 Driven Oxidoreduction Represents a Chemotherapeutic Target for HIV-1 Entry Inhibition

**DOI:** 10.1371/journal.pone.0147773

**Published:** 2016-01-27

**Authors:** Kathrin Reiser, Leen Mathys, Sophie Curbo, Christophe Pannecouque, Sam Noppen, Sandra Liekens, Lars Engman, Mathias Lundberg, Jan Balzarini, Anna Karlsson

**Affiliations:** 1 Karolinska Institute, Department of Laboratory Medicine, Division of Clinical Microbiology, F68, Huddinge, Sweden; 2 KU Leuven, Rega Institute for Medical Research, Minderbroederstraat 10, Leuven, Belgium; 3 Uppsala University, Department of Chemistry–BMC, BOX 576, Uppsala, Sweden; German Primate Center, GERMANY

## Abstract

**Background:**

The entry of HIV into its host cell is an interesting target for chemotherapeutic intervention in the life-cycle of the virus. During entry, reduction of disulfide bridges in the viral envelope glycoprotein gp120 by cellular oxidoreductases is crucial. The cellular thioredoxin reductase-1 plays an important role in this oxidoreduction process by recycling electrons to thioredoxin-1. Therefore, thioredoxin reductase-1 inhibitors may inhibit gp120 reduction during HIV-1 entry. In this present study, tellurium-based thioredoxin reductase-1 inhibitors were investigated as potential inhibitors of HIV entry.

**Results:**

The organotellurium compounds inhibited HIV-1 and HIV-2 replication in cell culture at low micromolar concentrations by targeting an early event in the viral infection cycle. Time-of-drug-addition studies pointed to virus entry as the drug target, more specifically: the organotellurium compound TE-2 showed a profile similar or close to that of the fusion inhibitor enfuvirtide (T-20). Surface plasmon resonance-based interaction studies revealed that the compounds do not directly interact with the HIV envelope glycoproteins gp120 and gp41, nor with soluble CD4, but instead, dose-dependently bind to thioredoxin reductase-1. By inhibiting the thioredoxin-1/thioredoxin reductase-1-directed oxidoreduction of gp120, the organotellurium compounds prevent conformational changes in the viral glycoprotein which are necessary during viral entry.

**Conclusion:**

Our findings revealed that thioredoxin-1/thioredoxin reductase-1 acts as a cellular target for the inhibition of HIV entry.

## Introduction

During the last 30 years, a variety of at least 26 anti-HIV drugs have been approved for clinical use. They target the virus at various stages of its life-cycle and can be grouped within the following categories: CCR5 antagonists, fusion inhibitors, nucleoside-, nucleotide- and non-nucleoside reverse transcriptase (RT) inhibitors, integrase inhibitors and protease inhibitors [1]. A combination of drugs that belong to these different categories is currently used for the highly active antiretroviral therapy (HAART), which is capable to cause a nearly complete inhibition of HIV replication. This allows the blood stream to be cleared from virus particles to levels under the detection limit and allows a partial restoration of the immune function [2]. However, HAART does not eradicate the virus from the patient’s body. Proviral DNA remains integrated within the genome of e.g. long-living HIV-infected resting CD4^+^ T-lymphocytes, where it remains latent until the treatment is discontinued [3]. Therefore, HAART is not a cure, but merely a treatment for HIV infection that needs to be sustained throughout the whole life of the HIV-infected individual. Even though the current treatment is effective and widely used, problems remain because of side-effects and the ability of the virus to become resistant to the drugs due to its intrinsic high mutation rate. It is estimated that 40–45% of HIV-infected individuals harbour drug-resistant virus strains with a rapidly increasing subgroup (5–10%) that exhibit resistance to most, if not all, classes of RT and protease inhibitors [4–6]. Hence, it is still important to identify novel targets and to further develop drugs that allow an even more successful treatment of HIV-infected individuals.

In search of novel classes of anti-HIV compounds, we have previously investigated the gold-containing compound auranofin and showed that it inhibits the reduction of the disulfide bonds in the viral glycoprotein gp120 by targeting thioredoxin reductase-1 (TrxR1) [7]. Auranofin is known to be a TrxR1 inhibitor and has been temporarily in clinical use for the treatment of rheumatoid arthritis [8]. The anti-HIV activity of auranofin was discovered when rheumatoid arthritis was treated in AIDS patients. The compound increased the CD4 counts in the HIV-infected patients, while the plasma HIV-RNA counts were lowered [9].

There are four major groups of TrxR1 inhibitors described in the literature that are or have been in clinical use or under investigation as potential therapeutic agents: gold-containing compounds, platinum-containing drugs, alkylating agents and dinitrohalobenzenes [10]. Whereas the gold-containing compounds have been used for the treatment of rheumatoid arthritis, the other groups of TrxR1 inhibitors are in use or under investigation for cancer chemotherapy [8, 11].

It has been shown that several cellular redox-regulating enzymes are involved in the entry of HIV-1 into its susceptible target cells [12–14]. These oxidoreductases are responsible for the reduction of disulfide bridges in gp120, following the interaction of gp120 with the cellular receptors CD4 and CXCR4/CCR5. The reduction of disulfide bridges in gp120 induces conformational changes in gp120 that enable the fusion of the cellular and viral membranes [15]. Cell-free and/or cell culture experiments have shown the involvement of three different oxidoreductases: protein disulfide isomerase (PDI), thioredoxin-1 (Trx1) and glutaredoxin-1 (Grx1) [12–14]. These enzymes are able to transmit electrons between proteins, thereby regulating the interconversion between disulfide bridges and free thiol groups. For this electron transfer, the enzymes are dependent on electron donors such as reduced glutathione and TrxR1. The latter is mainly responsible for the supply of electrons to Trx1, and to a lesser extent also to PDI. TrxR1 is a homodimeric flavoprotein that occurs predominantly in the cellular cytosol [16]. Each subunit has a molecular weight of about 55 kDa and contains an FAD and an NADPH binding site. In addition, it contains two redox-active sites: a conserved CVNVGC motif and a sequence containing the GCUG motif, in which U represents a selenocysteine [17]. In order to reduce disulfide bridges in substrate proteins, electrons are transferred from NADPH to FAD, and subsequently from the CVNVGC motif to the GCUG motif of TrxR1. Then, the electrons are transferred to Trx1 or other targets such as PDI. Reduced Trx1 (or PDI) can transfer these electrons to gp120, thereby reducing a disulfide bridge into two thiol groups. In contrast to TrxR1, Trx1 and PDI can occur extracellularly [18, 19]. They are associated with the cell surface by non-covalent interactions with integral membrane proteins, lipids or glycans.

TrxR1 might be an interesting target for the development of a novel functional class of anti-HIV drugs. By inhibiting TrxR1, the supply of electrons to Trx1 and PDI might be blocked, thereby compromising the reduction of gp120, which is crucial for HIV-1 entry. TrxR1 inhibitors may also have additional targets in the life-cycle of HIV that have not yet been properly investigated.

In this study, we focused on organotellurium compounds that were initially developed as TrxR1 inhibitors and investigated whether they can disturb the balance in the Trx1/TrxR1 redox system in cancer cells [20–22]. More than 20 organotellurium TrxR1 inhibitors have now been examined for their anti-HIV activity in CEM cell cultures and four were selected for further investigations. The examined TrxR1 inhibitors proved inhibitory against HIV-1 and HIV-2 in cell culture and this activity was due to the inhibition of an early stage event in the life-cycle of the virus. Our results point to inhibition of viral entry due to the inhibition of the Trx1/TrxR1-driven oxidoreduction of the viral envelope glycoproteins as their most likely mechanism of antiviral action.

## Material and Methods

### Materials

The compounds 6-(butyltelluro)-6-deoxy-β-cyclodextrin (TE-2), bis[4-(*N*,*N*-di(2-carbomethoxyethyl)amino)phenyl]telluride (TE-10), *N*,*N*-dimethyl-4-aminophenyl 3-phenoxypropyl telluride (TE-14) and 3-(butyltelluro)propanesulfonic acid sodium salt (TE-20) were synthesised as described previously [20–22], dissolved in dimethylsulfoxide (DMSO) at 25 mM and kept at -20°C until use, but not longer than 1 month.

The following cell lines were obtained through the NIH AIDS Reagent Program, Division of AIDS, NIAID, NIH: ACH-2 from Dr. Thomas Folks [23, 24], Sup-T1 from Dr. Dharam Ablashi, HHV-6 Foundation [25] and TZM-bl from Dr. John C. Kappes, Dr. Xiaoyun Wu and Tranzyme Inc. [26–30]. Human CD4^+^ T-lymphocytic CEM and C8166 cells were obtained from the American Type Culture Collection (ATCC) (Rockville, MD) and MT-4 cells were kindly provided by L. Montagnier (Pasteur Institute, Paris, France) [31]. Persistently infected HuT-78 cells (HuT-78/HIV-1) were obtained by exposure of HuT-78 cells (ATCC) to wild-type HIV-1(III_B_) during 3–4 weeks.

Buffy coat preparations from healthy, anonymous donors were obtained from the Blood Transfusion center in Leuven, Belgium. Peripheral blood mononuclear cells (PBMCs) were isolated using density gradient centrifugation with Lymphoprep (d = 1.077 g/ml) (Nycomed, Oslo, Norway). These cells were stimulated with 2 μg/ml phytohaemagglutinin (PHA; Sigma-Aldrich, Diegem, Belgium) and 5 U/ml IL-2 (Roche, Brussels, Belgium) and were then incubated at 37°C. After 3 days, the cells were washed and further incubated in the presence of 10 U/ml IL-2.

Recombinant HIV-1 protease was purchased from AnaSpec Inc. (Fremont, CA), recombinant HIV-1(III_B_) glycoprotein gp120 (CHO) from Immuno Diagnostics, Inc. (Woburn, MA) and Trx1 and TrxR1 from IMCO Corporation (Stockholm, Sweden) or Sino Biological Inc (Zoersel, Belgium). PMA (12-phorbol-13-myristate acetate), NADPH, biotin-maleimide, streptavidine-ALP, p-nitrophenyl phosphate, dextran sulphate (DS-8000, average weight 8,000 Da), 3’-azido-2’,3’-dideoxythymidine (AZT) and ritonavir were obtained from Sigma-Aldrich (Stockholm, Sweden). Alternatively, AZT was also synthesized by prof. Christophe Pannecouque, according to the method developed by Horwitz *et al*. [32], and ritonavir was also purchased from Molekula (München, Deutschland). T-20 (Fuzeon) was ordered from Roche (Brussels, Belgium). The mannose-specific plant lectin *Hippeastrum hybrid* agglutinin (HHA) was kindly provided by E. J. M. Van Damme (Ghent, Belgium) [33]. Auranofin was obtained from Alexis Biochemicals (Lausen, Switzerland), AMD3100 from AnorMed (Langley, British Colombia, Canada) and MagicMark XP Western Protein Standard from Novex (Ghent, Belgium). The antibodies MT17R6 (catalogue # 3580–3, anti-human Thioredoxin-1, mouse MAb; 0.5 mg/ml) and MT13X3-biotin (catalogue # 3580–6; anti-human Thioredoxin-1, biotinylated, mouse MAb, 0.5 mg/ml) were a kind gift from Mabtech (Nacka Strand, Sweden). The antibody AB9044 (anti-HIV p24 antibody, mouse MAb, clone number 38/8.7.47, Batch # GR145191-4, 1.2 mg/ml) was purchased from Abcam (Antwerp, Belgium).

### Inhibition of HIV-1 replication and cytostatic activity assays

The antiviral activity and toxicity of TE-2, TE-10, TE-14 and TE-20 were investigated in 2 settings, using either transformed laboratory cell lines or primary PBMCs.

To study the antiviral activity in transformed cells, CEM cells were suspended in fresh culture medium and infected with HIV-1(III_B_) or HIV-2(ROD) at 100 x CCID_50_ (cell culture infective dose 50%) per ml of cell suspension. Infected cell suspension (100 μl; 3 x 10^5^ cells/ml) was transferred to a 96-well microplate, mixed with 100 μl of the appropriate dilutions of compounds, and further incubated at 37°C. After 4 days, giant cell formation was recorded microscopically and the number of giant cells was estimated microscopically as percentage of the number of giant cells present in the non-treated virus-infected cell cultures.

The cytostatic activity assay using transformed cell lines was also performed in 96-well microtiter plates. 5–7.5 x 10^4^ human CD4^+^ T-lymphocyte CEM or human cervix carcinoma HeLa cells and a given amount of the test compounds were added to each well. The cells were allowed to proliferate for 72 h (96 h for HeLa) at 37°C in a humidified CO_2_-controlled atmosphere. At the end of the incubation period, the cells were counted in a Coulter counter (type ZM, Coulter Electronics, Analis, Ghent, Belgium).

To test the antiviral activity in fresh, PHA-stimulated PBMCs, an experiment was performed as previously described [34, 35]. Briefly, the cells were preincubated with HIV-1(III_B_), HIV-1(BaL) or HIV-1(HE) for 1 h at 37°C, using a viral load of 1000 x CCID_50_ per ml. Then, cell suspensions containing virus and 2 x 10^5^ PBMCs were seeded in a 96-well microtiter plate and a serial dilution of compound was added, in a total volume of 200 μl. After 4 days, the cells were passaged and fresh culture medium, containing IL-2 and compound, was added. Three days later, cellular supernatants were harvested and viral replication in the presence of the compounds was quantified using a p24 enzyme-linked immunosorbent assay (ELISA) (Perkin Elmer, Brussels, Belgium). The half maximal effective concentration (EC_50_) was defined as the compound concentration that is required to block viral replication by 50%.

A similar experiment was performed to study the toxicity of the compounds in fresh, PHA-stimulated, PBMCs. Mock-infected PBMCs (2 x 10^5^ cells) were exposed to a serial dilution of compound, in a total volume of 200 μl. Cells were passaged after 4 days. Cell survival and proliferation was assessed at day 7 using trypan blue-based cell counting with the Countess Automated Cell Counter (Invitrogen, Ghent, Belgium). The IC_50_ was defined as the concentration of the compound that inhibited cell proliferation by 50%.

### Virus expression

Persistently HIV-1-infected ACH-2 cells (1 x 10^6^ cells/ml) were cultured in the presence of 100 nM PMA. After three days, the cell culture supernatant was collected, cleared by centrifugation at 300 x g for 10 min and passed through a 0.45 μm filter. If indicated, the virus particles were further purified and concentrated using 200 μl supernatant and 50 μl magnetic beads from the μMACS VitalVirus HIV Isolation Kit (Miltenyi Biotech, Lund, Sweden) according to the manufacturer’s manual. The virus particles were released from the column by lysis using 2 x 80 μl of the provided lysis buffer containing 0.5% Igepal-630.

The HIV p24 content was measured in an Architect i2000SR (Abbott Laboratories, IL) as a measure of virus content and supernatant was either used directly or frozen at -80°C for later usage.

### Infectivity assay

TZM-bl cells (1 x 10^5^) were infected with diluted virus suspension (see virus expression) in a total volume of 0.2 ml DMEM medium in the presence of the compounds or an inhibitor control (nevirapine). After 40 h at 37°C, the luminescence was assayed and quantified in a Luminoskan Ascent (Thermo Labsystems, Schwerte, Germany) for luciferase activity using the OneGlo luciferase assay system (Promega, Nacka, Sweden).

To measure the infectivity of particles produced in the presence of the compounds, washed persistently HIV-1-infected ACH-2 cells (1 x 10^6^ cells/ml) were cultured with 100 nM PMA in the presence of the indicated concentration of compounds. After 3 days, the p24 content of the clarified supernatant (see virus expression) was measured. The cell supernatant was then used for an infectivity test (see infectivity assay) without the further addition of any compound to the cultures. The final dilution of the ACH-2 supernatant in the infectivity test was 1:100. A control experiment was performed, in which virus particles produced without the addition of compound, were used for the infectivity test. Additionally, the same amount of compound that could have been carried over with the virus containing supernatant in the original experiment was added to the infectivity test (final concentration: 1/100 of the indicated amount in the viral growth culture).

### Reverse transcriptase (RT) inhibition

The ability of the compounds to inhibit the activity of viral RT was determined by a Lenti RT Activity kit (Cavidi, Uppsala, Sweden) according to the manufacturer’s instructions. A poly-A-coated plate was incubated with the reaction bufferfor up to 1 h at 33°C. Then, a mixture of the virus preparation (see virus expression) and 25 μM of the test compounds or an inhibitor control (nevirapin) were added to the RT assay mixture and incubated overnight at 33°C. The plate was washed, exposed to the provided tracer for 90 min at 33°C and washed again, before the provided substrate was added to quantify the RT reaction that took place. The plate was incubated in the dark for 30 min and subsequently read at 405 nm in a Labsystem Multiskan RC (Artisan Technology Group, Champaign, IL, USA).

### Time-of-drug-addition assay

Two time-of-drug-addition (TOA) experiments were performed and adopted from previously reported methods [36, 37]. Briefly, MT-4 cells were infected with HIV-1(III_B_) at a multiplicity of infection of 0.5. Following a 1 hour adsorption period, cells were washed, distributed in a 96-well tray at 100,000 cells per 200 μl per well and incubated at 37°C.

For the first experiment, test compounds were added to the infected cell cultures at different time points (i.e. 0, 1, 2, 3, 4, 5, 6, 7, 8, 9, 24, and 25 h) after the start of infection.

In a second experiment, the resolution of the assay was increased by evaluating early time points in more detail. Therefore, the test compounds were added to the infected cell cultures at the following time points after the start of infection: 0, 15, 30, 45, 100 and 180 min; and 24 as well as 25 h.

Product added at time points 0, 15, 30 and 45 min was present at the start of the viral infection and washed away after 1 hour. After washing, fresh product was added to maintain viral suppression throughout the remaining of the experiment.

HIV-1 production was determined at 31 h post infection via a p24 ELISA (Perkin Elmer, Brussels, Belgium). The CXCR4 antagonist AMD3100 was used at 625 nM, dextran sulphate (DS-8000, an HIV adsorption inhibitor) at 12.5 μM, AZT (RT inhibitor) at 1.9 μM, ritonavir (HIV protease inhibitor) at 277 nM, T-20 (fusion inhibitor) at 4.5 μM and compound TE-2 at 50 μM, 25 μM or 12.5 μM.

### Inhibition of TrxR1-dependent reduction of HIV-1 gp120

After coating 96-well Maxi-Sorp ELISA plates (Nunc Inc, Roskilde, Denmark) with 100 μl gp120 in PBS (2 μg/ml), these were blocked with PBST (phosphate buffered saline, pH 7.4 with 0.05% Tween 20) as described before [14]). The experiment was performed with two different set-ups for each compound, but each reaction mixture contained 1 μM Trx1 + 100 nM TrxR1 + 240 μM NADPH + 100 μM of the compounds, with auranofin as an inhibition control. In the first set-up (TrxR1 + Trx1), the reaction mixtures were pre-incubated for 15 min at 37°C in the absence of the compounds, which were added after the preincubation period. In the second set-up (TrxR1 + compounds), the reaction mixtures were exposed to the different compounds, but in the absence of Trx1, which was added after the 15 min pre-incubation period. The reaction mixtures were then added to the gp120-coated wells to allow the reduction of gp120, incubated for 15 min at 37°C and then washed four times with PBST to remove any unbound material. Subsequently, the wells were incubated with 2 μM biotin-maleimide for 30 min at room temperature, then washed and incubated with steptavidine-ALP (diluted 1:1000) at room temperature. After 30 min incubation, the wells were washed with PBST and 1 mg/ml p-nitrophenyl phosphate dissolved in 10% diethanolamine pH 9.8 with 0.5 mM MgCl_2_ was added. The absorbance at 405 nm was measured using a microplate reader (Wallac Perkin-Elmer).

### Surface plasmon resonance (SPR) analysis

SPR analysis was performed to evaluate binding of TE-2 to immobilized recombinant TrxR1, PDI, gp120, gp41 and sCD4. Therefore, these ligands were immobilized on a CM5 sensor chip (GE Healthcare, Uppsala, Sweden) using standard amine coupling in the presence of 10 mM sodium acetate, pH 5. A high ligand-density chip was created, with ligand densities of 6759, 5004, 4119, 2900 and 5569 RU, respectively. A reference flow cell was used to control for non-specific binding and refractive index changes. All interactions were studied at 25°C on a Biacore T200 instrument (GE Healthcare, Uppsala, Sweden) in HBS-P (10 mM HEPES, 150 mM NaCl and 0.05% surfactant P20; pH 7.4) supplemented with 10 mM CaCl_2_ and 5% DMSO. TE-2 was tested at the concentrations 10, 50 and 250 μM. Samples were injected during 2 minutes at a flow rate of 30 μl/min, followed by a dissociation phase of 2 minutes. Each injection was followed by a regeneration step with 50 mM NaOH (injected during 10 seconds at a flow rate of 100 μl/min).

### Co-cultivation assay

Persistently infected HuT-78/HIV-1 cells were thoroughly washed to eliminate free virions. Next, 1 x 10^5^ HuT-78/HIV-1 cells were co-cultivated with 1 x 10^5^ Sup-T1 cells in 96-well plates (200 μl/well) during 20 h at 37°C, in the presence or absence of a serial dilution of compound. Afterwards, the formation of syncytia was evaluated microscopically. The EC_50_ was defined as the compound concentration required to suppress syncytia formation by 50%.

### Protease inhibition

The ability of the compounds to inhibit the activity of recombinant HIV-1 protease was determined by a SensoLyte 520 HIV-1 protease assay kit (AnaSpec Inc., Fremont, CA) according to the manufacturer’s instructions. Compounds (10 μl of a 250 μM, solution, giving a final concentration of 25 μM in the reaction mixture) and 40 μl HIV-1 protease (100 ng/well; AnaSpec Inc.) were added to a dark-bottom 96-well plate. Pepstatin A was included as an inhibitor control. The plate as well as the HIV-1 protease substrate solution (provided) were incubated for 15 minutes at 37°C and 50 μl protease substrate was added to all wells. The fluorescence was measured at Ex/Em = 490 nm/520 nm with a Varioskan Flash (Thermo Electron Corporation, Schwerte, Germany).

In order to confirm the inhibitory effect of TE-10, an additional assay was performed to study the inhibition of the HIV-1 protease by TE-10. Therefore, 2 x 10^5^ persistently infected HuT-78/HIV-1 cells were washed thoroughly and seeded in 48-well plates, in the presence or absence of compound. As reference compounds, the RT inhibitor AZT (3.7 μM) and the protease inhibitor ritonavir (2.8 μM) were used, as negative and positive controls, respectively. TE-10 was used at 1 μM and 20 μM. After 43 h of incubation at 37°C, virus-containing cell culture supernatants were harvested and the cells were removed by centrifugation (10 min at ± 900 x g). Then, virus was precipitated from the cell supernatant by centrifugation at ± 3.5 x 10^4^ x g for at least 2 h. The viruses were resuspended in PBS and lysed with Triton-X100 (Sigma-Aldrich, Diegem, Belgium). These viral lysates were then subjected to SDS-PAGE and subsequent Western blotting to detect the presence of p24 and its precursor p55, using an anti-p24 antibody (AB9044; final concentration 2.4 μg/ml).

### Western Blot analysis to detect thioredoxin and thioredoxin reductase in HIV-1 particles

Human CD_4_^+^ T-lymphocyte C8166 cell cultures were infected with HIV-1(NL4.3). After approximately 4 days, when abundant giant cell formation was microscopically visible, the HIV-1-infected cell culture was centrifuged at 1,000 rpm for 15 min at 4°C to remove cells and cell debris. To the supernatant was then added sucrose to reach a final concentration of 20%. The supernatant was centrifuged for 3 hours at 20,000 rpm in a high-speed Sigma 330K centrifuge. The virus pellet was washed with PBS containing 20% sucrose and centrifuged again for 3 hours at 20,000 rpm. After this centrifugation step, the virus pellet was washed a last time with PBS (without sucrose) and centrifuged for 3 hours at 20,000 rpm. The virus pellet was subsequently lysed with RIPA lysis and extraction buffer (Thermo scientific, Leuven, Belgium). The equivalent of 500 ng of p24 protein was loaded onto a 4–12% Bis-Tris PAGE gel (Invitrogen, Ghent, Belgium). After blotting to a PVDF membrane (GE Healthcare), Trx1 and TrxR1 were detected using the antibodies 10384-RP01 (Sino Biological Inc., final concentration 9.3 μg/ml) and 13093-RP01 (Sino Biological Inc., final concentration 9.3 μg/ml), respectively. MagicMark XP Western Protein Standard (Novex) was used to verify protein molecular weights. As positive controls, recombinant TrxR1 (13093-H07E, Sino Biological Inc.) and Trx1 (10384-HNAE, Sino Biological Inc.) were used.

### Quantification of Trx1 in viral particles produced in the presence of organotellurium compounds by ELISA

Persistently HIV-1-infected ACH-2 cells (1 x 10^6^ cells/ml) were cultured with 100 nM PMA and 25 μM organotellurium compound or an equivalent volume of DMSO (up to 0.1% of cell culture volume) as a negative control. After 3 days, the viral particles were isolated and lysed with the μMACS VitalVirus Isolation kit. The p24 content of the viral lysate was then quantified by ELISA. The lysates were also used to perform a quantitative Trx1 ELISA: 96-well Maxi-Sorp ELISA plates (Nunc Inc, Roskilde, Denmark) were coated with 100 μl MT17R6 (anti-Trx1 ab) (Mabtech) in carbonate buffer (2 μg/ml) and blocked with incubation buffer (Mabtech). The wells were washed five times with PBST and subsequently incubated at room temperature for 2 h with 100 μl of a 1:2 dilution of the viral lysates in ready-to-use assay buffer (Mabtech). The plates were then washed five times with PBST and incubated with 100 μl 12.5 ng/ml MT13X3-biotin (Mabtech) at room temperature for 2 h. After five washes with PBST, 100 μl steptavidine-ALP (diluted 1:1000) was added and incubated at room temperature. After 1 h incubation and an additional round of five washes with PBST, 1 mg/ml p-nitrophenyl phosphate dissolved in 10% diethanolamine pH 9.8 with 0.5 mM MgCl_2_ was added to each well. The absorbance at 405 nm was measured using a microplate reader (Wallac Perkin-Elmer). The results for the measured amounts of Trx1 associated to the virus particles were normalised to the measured amounts of p24.

### Statistical analysis

Statistical analysis was performed with the two-tailed, paired Student’s t-test using Microsoft Excel. P-values are indicated if significant (*: p < 0.05; **: p < 0.01; ***: p < 0.001).

## Results

### The organotellurium compounds are endowed with anti-HIV activity

**Fig 1:**
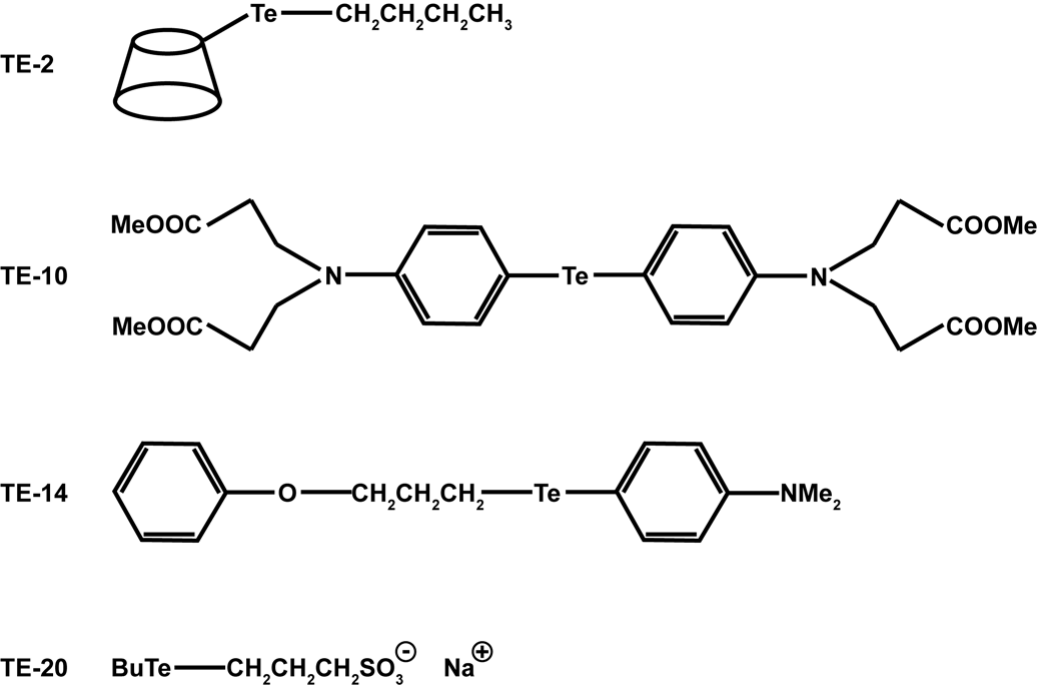
Structure of organotellurium compounds.

A broad range of more than 20 organotellurium TrxR1 inhibitors were evaluated for their inhibitory activity against HIV-1 and HIV-2 replication in CEM cell cultures by assessing the inhibition of virus-induced syncytia formation. Most of the compounds showed an activity against the laboratory HIV strains HIV-1(III_B_) and HIV-2(ROD) at concentrations lower than their antiproliferative concentrations. Fig 1 and Table 1 show the four organotellurium compounds that were selected for further investigation, as based on their previously known structural properties, and their pronounced anti-HIV and (limited) cytostatic activity. Compounds TE-2, TE-14 and TE-20 were antivirally active in the low micromolar range (EC_50_: 0.86 to 2.9 μM), whereas TE-10 was approximately 10-fold less inhibitory (EC_50_: 15–16 μM). Compound TE-2 was virtually not cytostatic against proliferating human CD4^+^ T-lymphocyte CEM and cervix carcinoma HeLa cell cultures (half maximal inhibitory concentration (IC_50_) > 100 μM) and therefore, TE-2 was endowed with the highest antiviral selectivity (SI = IC_50_/EC_50_: 167–227) among the investigated compounds (Table 1).

**Table 1:** Anti-HIV and cytostatic activities of organotellurium compounds in CEM and HeLa cell cultures.

Compound code	Chemical name	MW (g/mol)	Hydro-phobicity	EC_50_ (μM) ^a)^ HIV-1 (III_B_)	EC_50_ (μM) ^a)^ HIV-2 (ROD)	IC_50_ (μM) ^b)^ CEM	IC_50_ (μM) ^b)^ HeLa	Ratio IC_50_/EC_50_ HIV-1 (CEM)	Ratio IC_50_/EC_50_ HIV-2 (CEM)	Reference
TE-2	6-(butyltelluro)-6-deoxy-β-cyclodextrin	1427.68	Hydrophilic	1.1 ± 0.25	1.5 ± 0.52	≥ 250	205 ± 40	227	167	[22]
TE-10	bis[4-(*N*,*N*-di(2-carbomethoxyethyl)amino)phenyl] telluride	656.19	Hydrophobic	16 ± 7.9	15 ± 2.6	≥ 250	128 ± 29	16	17	[21]
TE-14	*N*,*N*-dimethyl-4-aminophenyl 3-phenoxypropyl telluride	382.95	Hydrophobic	0.86 ± 0.21	1.4 ± 0.56	22 ± 2	25 ± 4	26	16	[21]
TE-20	3-(butyltelluro)-propanesulfonic acid sodium salt	329.9	Hydrophilic	2.7 ± 2.6	2.9 ± 1.3	103 ± 27	101 ± 3	38	36	[20]

^a)^ 50% effective concentration or concentration required to protect CEM cells against the cytopathogenicity of HIV by 50%

^b)^ 50% inhibitory concentration or compound concentration required to inhibit three-day drug-exposed CEM or HeLa cell proliferation by 50%.

Data were obtained in two to three independent experiments. The mean values ± SD are shown.

The activity and toxicity of TE-2, TE-10, TE-14 and TE-20 were also evaluated using freshly isolated, HIV-1-infected, PHA-stimulated, primary PBMCs (Table 2). The antiviral inhibitory activities of TE-2, TE-14 and TE-20 were somewhat weaker in PBMC cultures, as compared to the ones observed in the CEM cell line, but TE-10 showed a comparable or slightly increased antiviral activity in PBMCs. It should be noticed that, in general, the compounds were not only active against the X4 HIV-1(III_B_), but also against the dual-tropic HIV-1(HE) and the R5 HIV-1(BaL) strains. The compound toxicities were more pronounced in seven-day drug-exposed PBMCs (Table 2) than in three-to-four-day drug-exposed CEM or HeLa cell cultures (Table 1).

**Table 2:** Anti-HIV and cytotoxic properties of organotellurium compounds in PHA-stimulated PBMCs.

Compound code	EC_50_ (μM)^a)^ HIV-1 (III_B_)	EC_50_ (μM)^a)^ HIV-1 (BAL)	EC_50_ (μM)^a)^ HIV-1 (HE)	IC_50_ (μM)^b)^ PBMC
TE-2	15 ± 7	≥ 25^c)^	6.6 ± 4.2	54 ± 16
TE-10	12 ± 4	12 ± 10	6.6 ± 2.1	19 ± 2
TE-14	4.9 ± 2.1	9.6 ± 10	5.4 ± 3	9.9 ± 2.5
TE-20	18 ± 12	≥ 25^d)^	11 ± 9	75 ± 53

Data were obtained by at least two to three independent experiments. The mean values ± SD are shown.

^a)^ 50% effective concentration or compound concentration required to protect PBMCs against the cytopathicity of HIV by 50%

^b)^ 50% inhibitory concentration or concentration required to inhibit the proliferation of seven-day drug-exposed PHA-stimulated PBMCs by 50%

^c)^ 20–40% inhibition at indicated concentration

^d)^ 30–50% inhibition at indicated concentration

The data also indicate that the anti-HIV efficacy of the organotellurium compounds is not simply dependent on their hydrophobicity because two of them have hydrophilic rather than hydrophobic properties.

### Effect of the test compounds on different stages in the viral life-cycle

**Fig 2:**
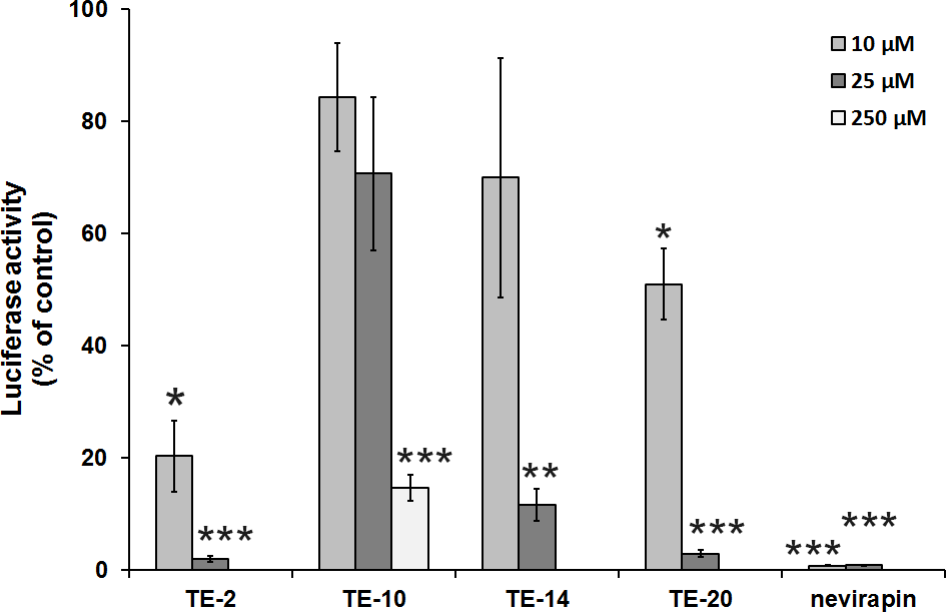
Inhibition of HIV infection of TZM-bl cell cultures. Monolayer TZM-bl cells were infected with HIV-1 in 0.2 ml DMEM medium in the presence of 10 μM or 25 μM test compound (in case of TE-10 also 250 μM) or an inhibitor control (nevirapin). Additionally, one well was mock infected (no virus, negative control). After 40 h, the cells were assayed for expression of luciferase activity. The value for the vehicle control was set to 100% in each individual experiment and the negative control was set to 0%. All other values were normalised against these reference values. The data are means of three independent experiments; the error bars represent the SEM. P-values are indicated if significant (*: p < 0.05; **: p < 0.01; ***: p < 0.001).

Several assays were used to determine the mechanism of antiviral action of the organotellurium compounds. First, the aim was to identify at which stage (early or late) of the viral life-cycle the compounds are antivirally active. With this aim, an infectivity assay was performed using TZM-bl (HeLa/CD4/CXCR4/CCR5) cells which contain a luciferase gene that is under control of the HIV-1 promoter. Upon viral entry, reverse transcription and proviral DNA integration into the host genome (early replication stage processes), luciferase is expressed. Therefore, the amount of luminescence is quantitatively correlated to the amount of integrated virus in the infected cell cultures. Once the virus is integrated, compounds that inhibit an early event in the replication cycle of the virus will have no effect on the luminescence signal anymore. All compounds inhibited the induction of the luminiscence signal upon HIV-1 incorporation in the cellular genome in a dose-dependent fashion (Fig 2). Compound TE-10 required an approximately 10-fold higher concentration for a similar inhibition of the luminescence signal than the other organotellurium compounds, which can be correlated to its approximately 10-fold higher EC_50_ value in the HIV-infected CEM cell cultures (Table 1). Nevirapine (a non-nucleoside RT inhibitor) showed full inhibition at 10 μM. Given the relatively short drug-incubation time to the confluent TZM-bl cell cultures (40 hours), the inhibition of virus replication is most likely due to a specific antiviral effect of the compounds. This is confirmed by our observations that confluent HeLa cell cultures exposed to the TE compounds for a time period of ~ 3 days, do not show any microscopically noticeable sign of cytotoxicity. The pronounced compounds’ effect on luminescence induction in the HIV-1-infected HeLa cell-derived TZM-bl cultures demonstrates that the organotellurium compounds dose-dependently inhibit virus replication at an early event in the infection cycle.

### The TrxR1 inhibitors do not inhibit HIV-1 reverse transcriptase activity

**Fig 3:**
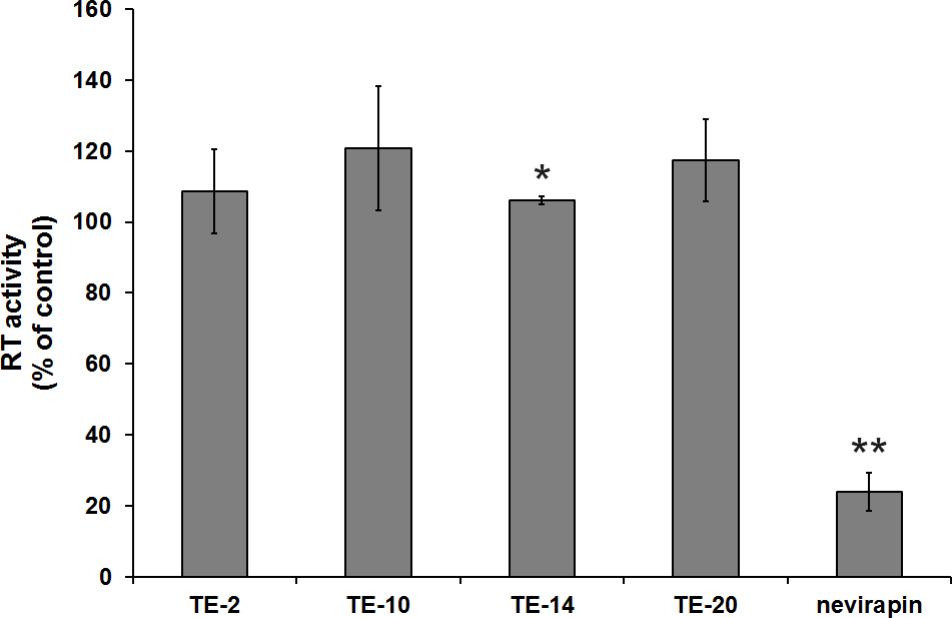
Inhibition of HIV-1 reverse transcriptase. A poly-A plate (Cavidi) was incubated with the reaction buffer and subsequently, the isolated virus sample together with 25 μM of the respective compound or nevirapin (inhibitor control) was added and incubated overnight. The plate was exposed to the provided tracer and the provided substrate was added. The plate was incubated in the dark for 30 min and the absorbance was read at 405 nm. The value for the vehicle control was set to 100% and all other values were normalised to this reference value. The data are the means of three independent experiments; the error bars represent the SEM. P-values are indicated if significant (*: p < 0.05; **: p < 0.01; ***: p < 0.001).

Since reverse transcription is one of the crucial steps during the early replication phase, the compounds were examined for their ability to inhibit HIV-1 RT using a commercially available kit. The results showed that none of the compounds was capable of inhibiting the RT polymerase activity at 25 μM (Fig 3). The positive control nevirapine produced pronounced, however not complete inhibitory activity at the 25 μM-concentration. The remaining activity in the presence of nevirapine might be due to the rather high number of virus particles in the assay providing an excess of viral RT and/or to the fact that the poly rA.dT template is not the optimal template for demonstrating anti-RT activity of NNRTIs such as nevirapine. Poly rC.dG has been demonstrated to generally provide better NNRTI inhibition efficacy than other templates (i.e. ~ 10-fold for nevirapine) [38]. Overall, the data indicate that, although the compounds seemingly target an early event in the viral infection cycle, they do not inhibit the viral RT.

### Time-of-drug-addition (TOA) reveals virus entry as target for viral inhibition

**Fig 4:**
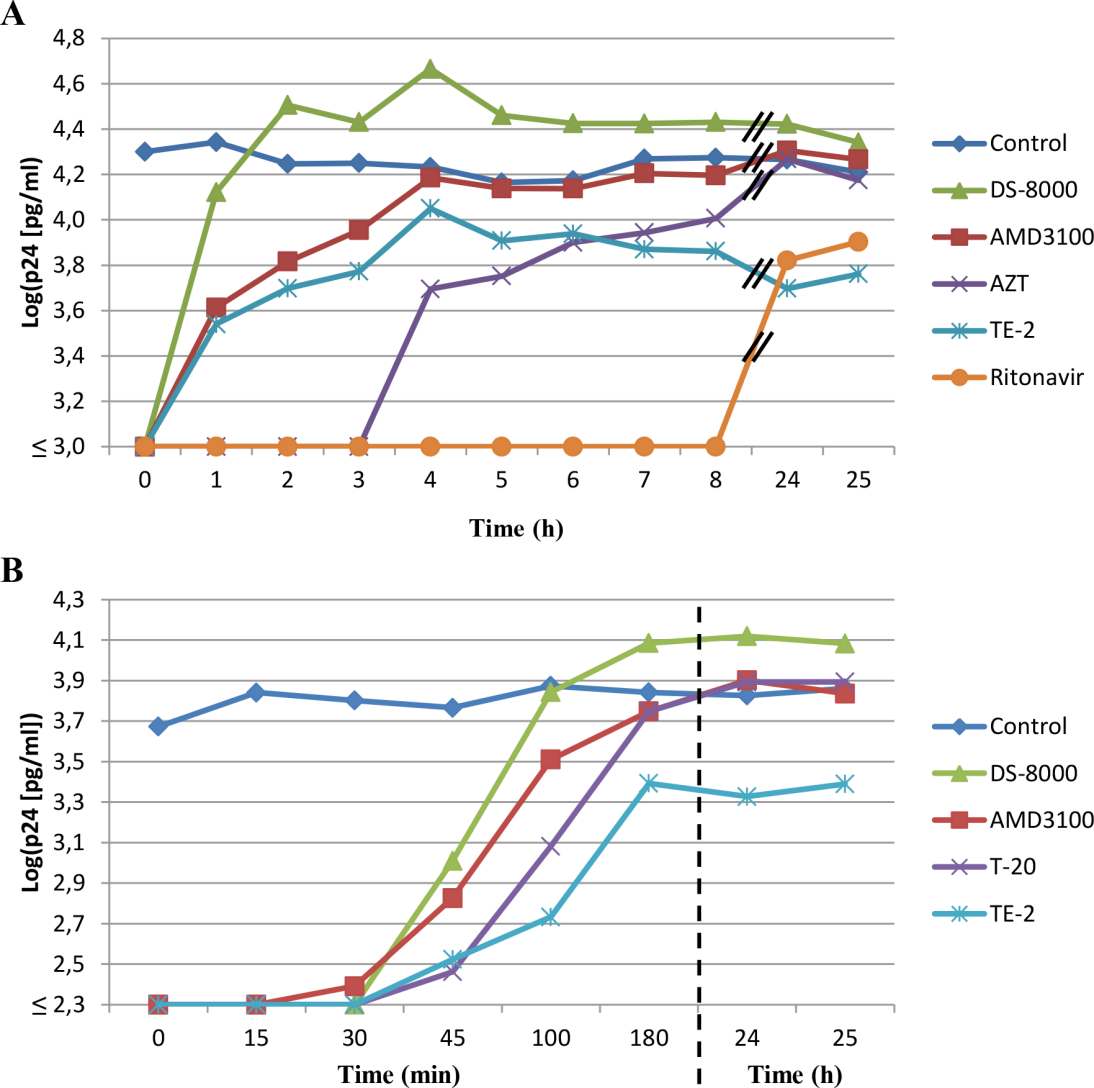
Time-of-drug-addition in HIV-1 infected MT-4 cell cultures. MT-4 cells were infected with HIV-1(III_B_) for 1 h after which the cells were washed and distributed in a 96-well plate. Test compounds were added at different time points after the start of infection, indicated on the abscis of the graphs (Panels A & B). Whereas in Panel A, the focus lies on a wide array of time points in the infection cycle of HIV-1, Panel B focuses on the very early time points during the viral replication cycle. 31 h after the start of the experiments, cellular supernatants were harvested and HIV-1 replication was quantified using a p24 ELISA. AMD3100 was used at 625 nM, DS-8000 at 12.5 μM, AZT at 1.9 μM, ritonavir at 277 nM, T-20 at 4.5 μM and compound TE-2 at 50 μM. The graphs are representatives of 2–3 independent experiments.

Since our search for the time of intervention of the compounds point to an early life-cycle event of the virus, TOA assays were performed with TE-2. This compound was chosen because of its selectivity index (SI > 100) allowing the addition of the compound to the cell cultures at a concentration nearly 50 times higher than its EC_50_ value (preferable for an optimal TOA experiment) [36, 37]. In a first TOA experiment, TE-2 (50 μM) was added to CD4^+^ T-lymphocyte MT-4 cell cultures at different time points after HIV-1 exposure (Fig 4A). Full antiviral effect of TE-2 was only present when the compound was added at the moment of HIV-1 infection (t = 0 h) and was already mostly lost when added at t = 1 h after infection. This is consistent with the effect of adsorption/entry inhibitors such as dextran sulphate (DS-8000) and the bicyclam CXCR4 antagonist AMD3100. A similar effect could also be observed at lower TE-2 concentrations such as 25 μM and 12.5 μM (data not shown). Addition of the HIV nucleoside RT inhibitor AZT and the protease inhibitor ritonavir could be delayed for at least 3 h and 8 h, respectively, after virus infection without losing their inhibitory activity (Fig 4A). Therefore, it could be concluded that TE-2 exerts its anti-HIV activity at the early viral entry process. Similar data were obtained with TE-14 added at 25 μM to the virus-infected cell cultures, although the inhibition of the virus production was less pronounced than the one observed for 50 and 25 μM of TE-2 (data not shown).

To refine the assay, a second TOA experiment was performed where shorter time intervals between product addition were applied. Fig 4B shows that the adsorption inhibitor DS-8000 and the CXCR4 antagonist AMD3100 lose their activity when administered 30–45 minutes post-infection. TE-2 showed a profile resembling that of the fusion inhibitor enfuvirtide (T-20) (Fig 4B). These data demonstrate that TE-2 most likely interferes with (a) viral infection step(s) resulting in, or coinciding with, the fusion of the cellular and viral membrane. The fact that virus titers in the TE-2-exposed cell cultures were somewhat lower than control through the whole incubation experiment may point to a slight toxic side effect of TE-2 at the administered drug concentration, rather than assuming a second mechanism of virus suppression by this compound. In fact, lower TE-2 concentrations did not show such virus-suppressive effects during the whole time period of the experiment.

### Inhibition of the reduction of disulfide bonds of gp120 by the test compounds

**Fig 5:**
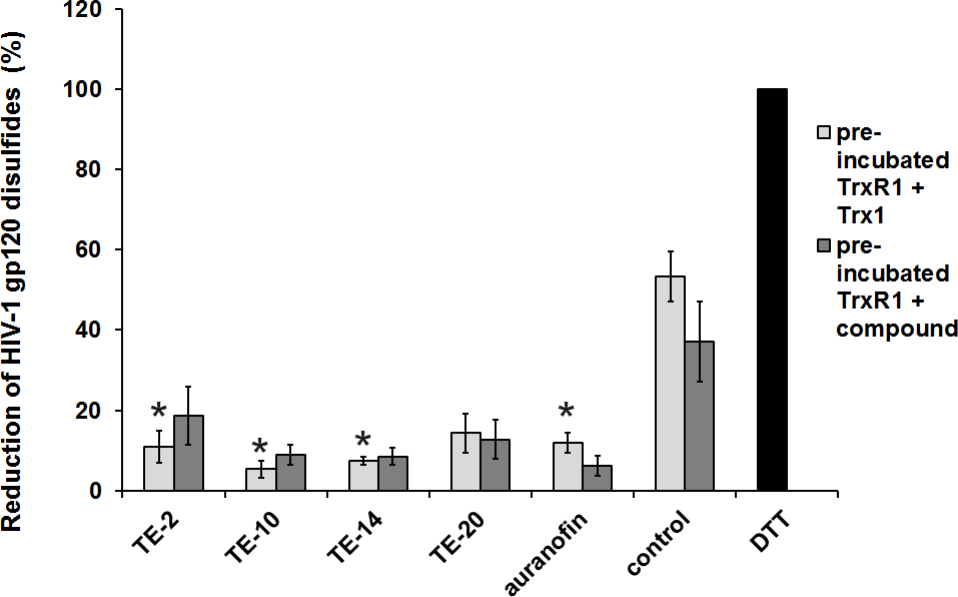
Inhibition of the reduction of gp120 in the presence of the test compounds. An ELISA was performed with two set-ups for each compound, the reference compound (auranofin), a vehicle control (DMSO) and DTT. In the first set-up (TrxR1 + Trx1)_preincub_., TrxR1 was shortly (15 min) pre-incubated with Trx1 before adding the compounds. In the second set-up (TrxR1 + compounds)_preincub_, TrxR1 was shortly (15 min) pre-incubated with the compounds before adding Trx1. The final reaction mixture contained 1 μM Trx1 + 100 nM TrxR1 + 240 μM NADPH + 100 μM compound or auranofin. DTT was used as a reduction control. ELISA plates were coated with recombinant human gp120. The reaction mixtures were added to the wells to allow reduction of the coated gp120, incubated for 15 min at room temperature and exposed to streptavidine-ALP and p-nitrophenyl phosphate. The value for the DTT was set to 100% in each individual experiment and all other values were normalised against this reference value. The data are means of three independent experiments; the error bars represent the SEM. P-values were calculated for each reaction in relation to its control and are indicated if significant (*: p < 0.05; **: p < 0.01; ***: p < 0.001).

The TOA experiments demonstrated that the organotellurium compounds (i.e. TE-2) are active at the viral entry stage of the replication cycle, probably at a step coinciding with, or resulting in, membrane fusion. This observation could be explained by the findings that CD4 binding induces disulfide oxidoreduction of gp120, thereby leading to conformational changes that enable membrane fusion [15]. Therefore, it was investigated whether the antiviral effect of the compounds could be due to an inhibition of gp120 reduction as has been previously proposed for auranofin [7]. The results (Fig 5) show that all organotellurium compounds have the capacity to inhibit the reduction of gp120 by TrxR1 in a cell-free setting. Dithiothreitol (DTT) was used as a strong reducing reference agent capable of opening a maximum number of disulfide bonds within gp120. In the control condition (Trx1 + TrxR1 + NADPH without compound) gp120 was reduced at 38–54% of the capacity of DTT (set at 100%). In the presence of 100 μM auranofin, reduction of the disulfide bonds was decreased to 6–10%. The organotellurium compounds diminished reduction of gp120 to 6–19% of the maximum value, depending on the structure of the compound. Thus, all compounds, including the reference compound auranofin, inhibited reduction of gp120 by Trx1 + TrxR1 to a considerable extent. No significant difference in the extent of inhibition was observed when TrxR1 was first shortly exposed to Trx1 before addition of the compounds (TrxR1 + compound; dark grey bars), or alternatively, when TrxR1 was first shortly exposed to the compounds before addition of Trx1 (TrxR1 + Trx1; light grey bars) (Fig 5).

### Binding affinity experiments

**Fig 6:**
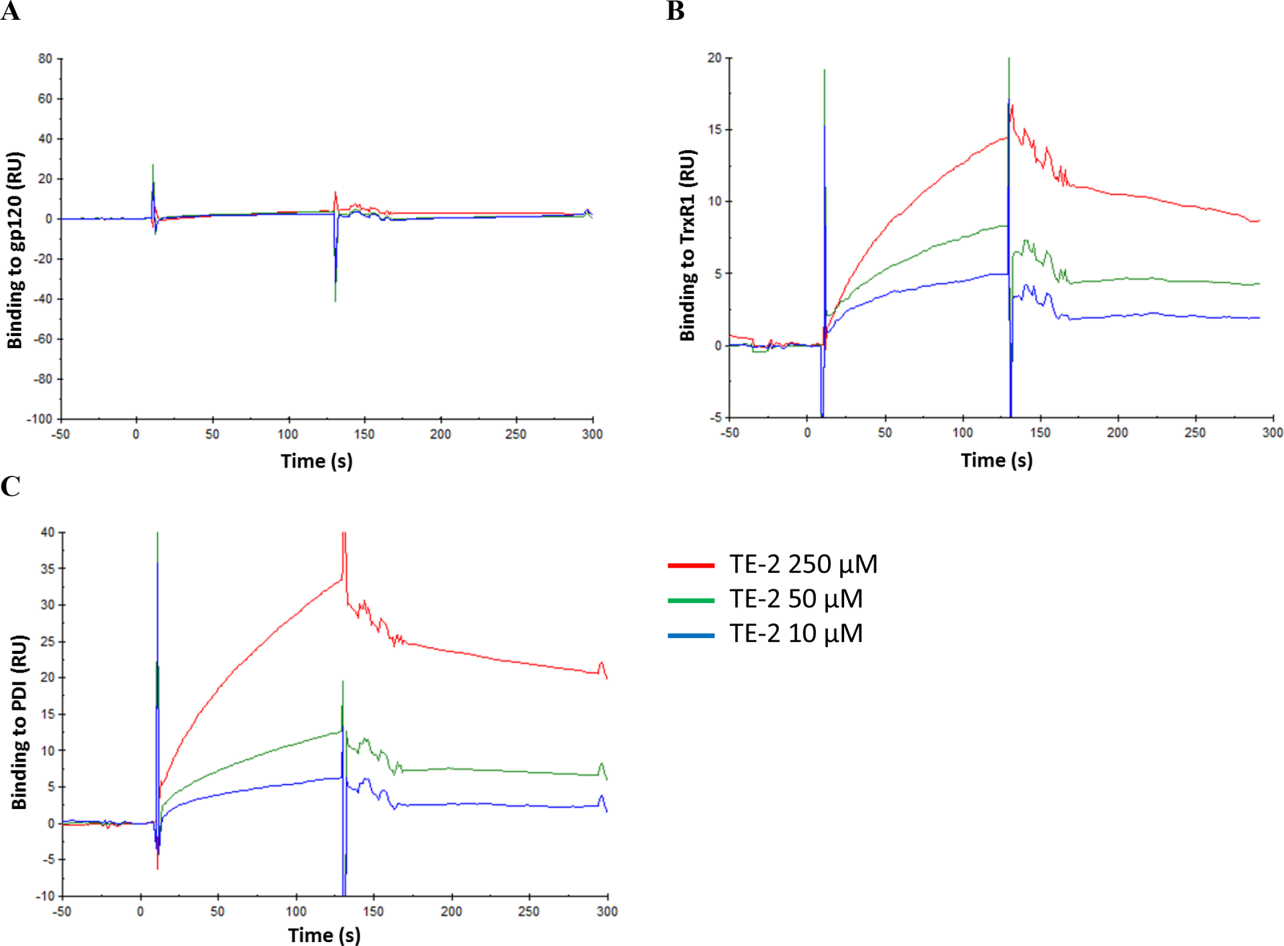
Binding of TE-2 to HIV-1 gp120, TrxR1 or PDI by SPR. The SPR technique was used to evaluate binding of 3 concentrations of TE-2 to gp120, TrxR1 and PDI, using a high density CM5 chip and a Biacore T200 instrument. The SPR sensorgrams show the binding of TE-2 to the immobilised ligand on the chip, and the subsequent dissociation (after 120 sec) of TE-2. (A) Binding of TE-2 to gp120 (B) Binding of TE-2 to TrxR1. (C) Binding of TE-2 to PDI.

To confirm the direct targeting of TrxR1 by the organotellurium compounds, surface plasmon resonance (SPR) experiments were performed studying the interaction of the TE-compounds with TrxR1. In addition, binding to CD4, gp120 and gp41 was also studied to rule out these (glyco)proteins as direct targets of the TE-compounds. It was shown that none of the organotellurium compounds had a measurable specific interaction with gp120, gp41, CD4 or the negative control human serum albumin (HSA), not even at concentrations up to 250 μM (Fig 6; not all data shown). Therefore we assume that none of the compounds block virus entry by directly interacting with the envelope proteins or with the cellular HIV receptor CD4. However, TE-2 was able to specifically bind TrxR1 (Fig 6B). By using various analyte (TE-2) concentrations, it was shown that the efficiency of this binding is concentration-dependent, indicating that the response seen in the SPR experiment was due to a specific interaction between TE-2 and TrxR1. TE-2 was also found to dose-dependently interact with PDI (Fig 6C). These data suggest that TE-2 targets cellular oxidoreductases (i.e. TrxR1), required for the conformational changes in gp120 upon viral infection of the target cells, rather than a virus-encoded (glyco)protein such as gp120 or gp41.

### Effect of the organotellurium compounds on syncytia formation in HuT-78/HIV-1 and Sup-T1 co-cultures

It has been shown earlier that a variety of compounds directly interacting with the HIV-1 envelope or cellular CD4/CXCR4/CCR5 (co)receptors (i.e. neutralising antibodies, carbohydrate-binding agents, enfuvirtide, the bicyclam AMD3100, maraviroc) are able to prevent syncytium formation in co-cultures of persistently HIV-1-infected HuT-78/HIV-1 cells and non-infected CD4^+^-Sup-T1 cells. It would be of interest whether the organotellurium compounds for which we have shown that they do not directly interact with gp120, gp41 or CD4 could have a similar inhibitory effect in such co-cultures. TE-2, TE-10, TE-14 and TE-20 were therefore investigated for their inhibitory potential against syncytium formation in co-cultures of persistently HIV-infected HuT-78/HIV-1(III_B_) cells and uninfected CD4^+^ T-lymphocyte Sup-T1 cells. In control cultures (without test compound) abundant syncytium formation was microscopically visible within 20 h after initiation of the co-cultures. None of the compounds was able to block syncytium formation at a concentration as high as 250 μM (Table 3). Auranofin also failed to inhibit syncytium formation in these cell cultures (data not shown). Instead, the CXCR4 antagonist bicyclam AMD3100, the carbohydrate-binding agent HHA and the gp41-binding enfuvirtide (T-20) inhibited syncytium formation in this assay at low micromomolar (AMD3100) or nanomolar (HHA and T-20) concentrations.

**Table 3:** Inhibition of syncytia formation in cocultures of HuT-78/HIV-1 and Sup-T1 cells.

Compound	IC_50_^a^ (μM)
TE-2	>250
TE-10	>250
TE-14	>250
TE-20	>250
HHA	0.016 ± 0.007
AMD3100	9.3 ± 4.3
T-20	0.275 ± 0.150

^a^50% Inhibitory concentration or compound concentration required to inhibit syncytium formation between persistently HIV-1-infected HuT-78 cells and uninfected SupT1 cells by 50% after ~ 24 hours of cell co-cultivation.

Data are derived from 2 independent experiments.

### Effect of the test compounds on the virus production and infectivity of newly formed virus particles in ACH-2 cell cultures

**Fig 7:**
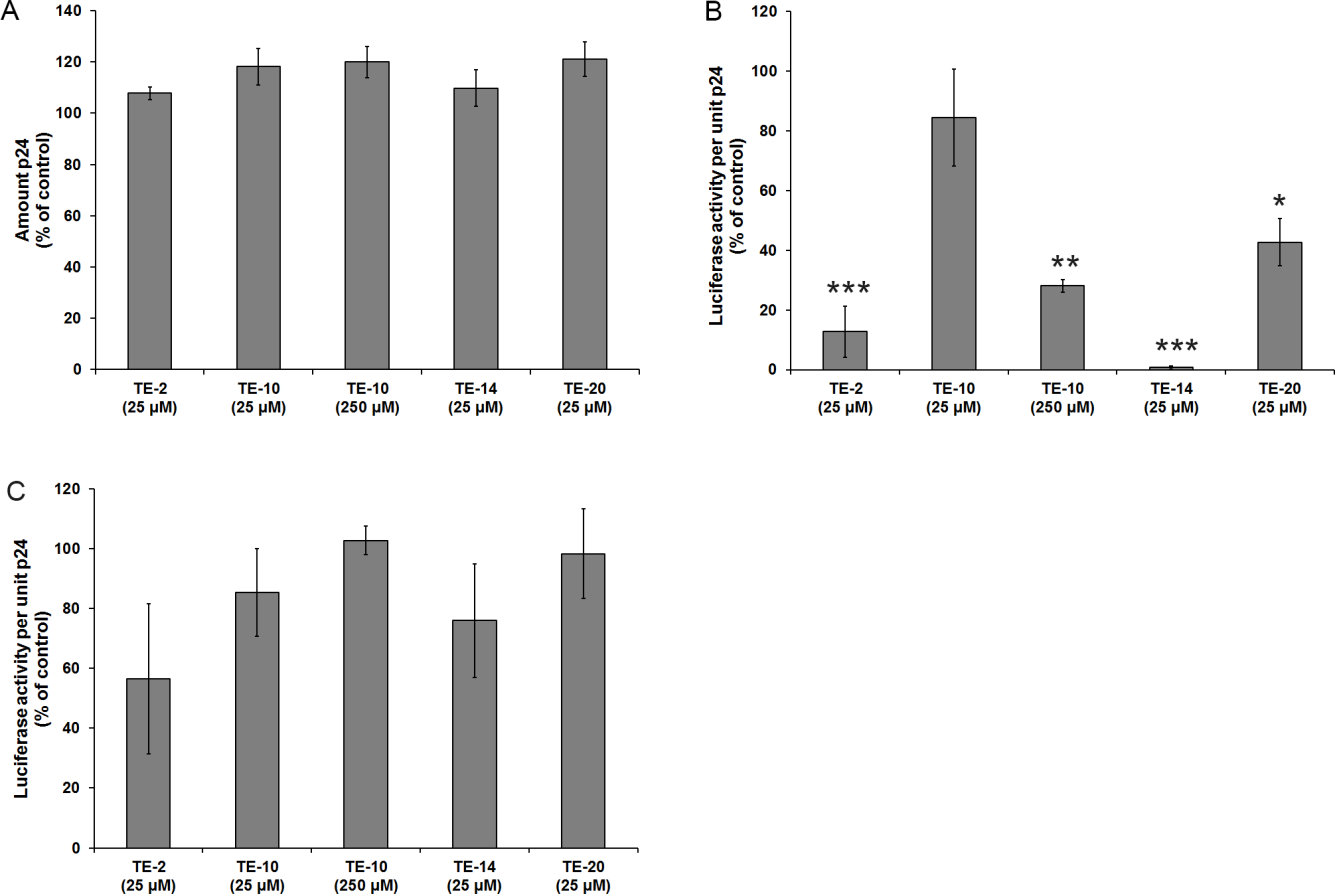
Effect of the organotellurium compounds on HIV-1 production in persistently HIV-1 infected ACH-2 cell cultures. (A) Amount of produced virus measured by p24 production: Persistently HIV-1-infected ACH-2 cells were cultured in the presence of 100 nM PMA and 25 μM of the indicated test compound (also 250 μM for TE-10). After 3 days, the supernatant was clarified and the amount of p24 was measured by ELISA. The value for the control was set to 100% in each individual experiment and all other values were normalised against these reference values. The data are means of three independent experiments; the error bars represent the SEM. P-values are indicated if significant (*: p < 0.05; **: p < 0.01; ***: p < 0.001).(B) Infectivity of the produced virus particles per unit p24: The clarified supernatant of the drug-treated ACH-2 cell cultures were further used for an infectivity test in TZM-bl cell cultures (without the addition of any compound). TZM-bl cells were infected with the diluted virus supernatants in a total volume of 0.2 ml DMEM (eventual dilution of virus supernatant: 1:100). After 40 h, the cells were assayed for luciferase activity. The value for the control was set to 100% in each individual experiment and all other values were normalised against these reference values. The data are means of three independent experiments; the error bars represent the SEM. P-values are indicated if significant (*: p < 0.05; **: p < 0.01; ***: p < 0.001).(C) Control experiment: A control experiment was performed, in which virus particles produced without the addition of compound, were used for an infectivity test. The same amount of compound that could have been carried over with the virus containing supernatant in the original experiment (Panel B) was added to the infectivity test (final concentration: 1/100 of the indicated amount in the viral growth culture). The value for the vehicle control (DMSO) was set to 100% in each individual repeat and all other values were normalised these reference values. The data are means of three independent experiments; the error bars represent the SEM. P-values are indicated if significant (*: p < 0.05; **: p < 0.01; ***: p < 0.001).

The effect of the presence of the TE-compounds on the production and the infectivity of viral particles newly formed in persistently HIV-1-infected ACH-2 cell cultures was investigated. Again, a luciferase-based assay was performed, but here the individual TE-compounds were not added during the infection of the TZM-bl cells, but during the growth of the persistently HIV-1-infected ACH-2 cells to reveal whether the compounds have an effect on the production and the infectivity of newly formed virus particles. The results show that the amount of p24 production and thus the amount of released virus particles was not affected by any of the compounds at 25 μM, or 100 μM for TE-10 (Fig 7A). These findings are in agreement with our earlier findings that HIV inhibition occurred at an early event in the viral infection cycle (Fig 2). However, there was a pronounced suppressive effect on the infectivity of the virus particles produced by the ACH-2 cell cultures in the presence of the test compounds. When the culture supernatants from the experiment shown in Fig 7A were titrated on TZM-bl cell cultures, the exposure to TE-2 and TE-14, and to a lesser extent to TE-10 and TE-20 of the persistently HIV-1-infected ACH-2 cell cultures resulted in the production of substantially less infectious virus particles (normalized for equal p24 levels) (Fig 7B). To show that this effect was not caused by residual compound that was carried over from the cell growth media into the luciferase assay, a control experiment was performed (Fig 7C), which revealed that the decreased infectivity shown in Fig 7B could not be explained by the presence of residual compound in the supernatants of the TE-10, TE-14 and TE-20-exposed persistently HIV-1-infected ACH-2 cell cultures.

### The TrxR1 inhibitors do not inhibit the viral protease activity

**Fig 8:**
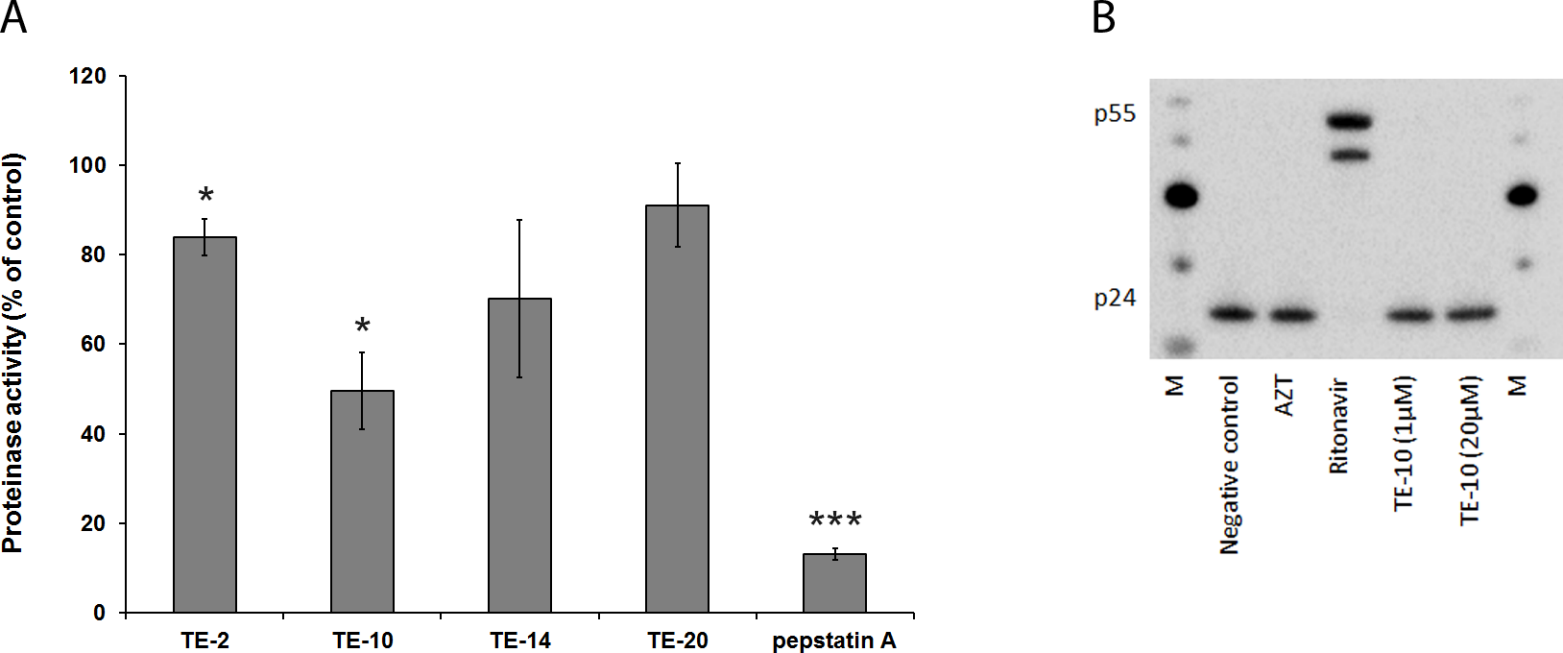
Inhibition of HIV-1 protease. (A) Inhibition of HIV-1 protease activity: The HIV-1 protease (100 ng/well) and the compounds (25 μM concentration) or an inhibitor control (pepstatin A) were added to a flat-bottom 96-well plate. The plate was incubated for 15 minutes at 37°C and the provided protease substrate was added to all wells. The fluorescence was measured at Ex/Em = 490 nm/520 nm. The value for the vehicle control was set to 100% in each individual experiment and all other values were normalised to this reference value. The data are means of three independent experiments; the error bars represent the SEM. P-values are indicated if significant (*: p < 0.05; **: p < 0.01; ***: p < 0.001).(B) Inhibition of HIV-1 protease activity in viral particles: Persistently infected HuT-78/HIV-1 cells were incubated in the presence or absence of compound (3.7 μM AZT, 2.8 μM ritonavir or 1μM/20μM TE-10). After 43 h, virus particles in the cellular supernatants were harvested, lysed and subjected to Western blotting. P24 and its precursor p55 were detected using an anti-p24 antibody. Negative control: no compound added during incubation. M: MagicMark XP Western Protein Standard.

One way of explaining the decreased viral infectivity of virus particles produced in the presence of the TE-compounds would be an inhibitory effect of these compounds on maturation, which is dependent on the viral protease activity. Therefore, the compounds were examined for their ability to inhibit the virale protease, using a commercially available kit. It was shown that none of the compounds markedly inhibited the protease activity at 25 μM, except for TE-10 which decreased the protease activity by 50% at 25 μM (Fig 8A). The positive controls pepstatin A produced a pronounced inhibitory activity at the 25 μM-concentration.

Since TE-10 seemed to have some inhibitory effect in the commercial HIV-1 protease assay (Fig 8A), an additional experiment was performed to reveal the relevance of this finding. Persistently HIV-1-infected HuT-78/HIV-1 cells were exposed to TE-10 or the reference compound ritonavir, during which time period new viral particles were produced. Initially, these virions will bear the precursor p55 protein that is cleaved by the protease to form p24. It was shown that the positive control ritonavir blocks this cleavage, enabling the abundant detection of p55 in viral lysates, using Western blotting (Fig 8B). In contrast, the negative control (lack of compound), AZT and TE-10 (exposed at 1 and 20 μM) were found to allow efficient p55 cleavage in the viral particles, resulting in the detection of p24 and the absence of intact p55 (Fig 8B). Taken together, we can conclude that the decreased infectivity of the virus particles derived from drug-exposed cell cultures is unlikely to be due to the inhibition of the virus-encoded protease.

### Detection of Trx1 and TrxR1 in the virus particles derived from virus-containing cell supernatants

**Fig 9:**
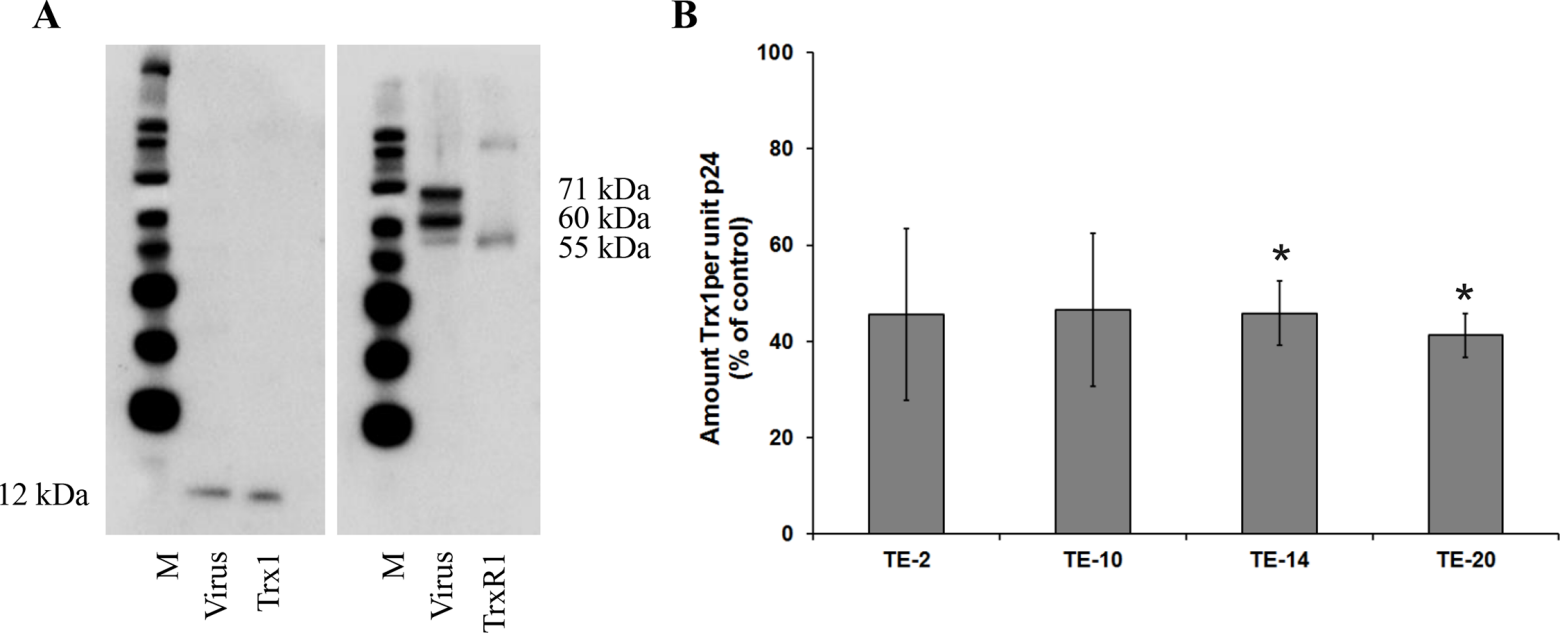
Prevalence of Trx1 and TrxR1 in HIV particles. (A) Western Blot analysis to detect Trx1 and TrxR1 in the envelope of HIV-1: HIV-1(NL4.3) particles (500 ng of p24 protein) were lysed and loaded onto a 4–12% Bis-Tris PAGE gel. Trx1 and TrxR1 were detected using the antibodies 10284-RP01 and 13093-RP01, respectively. As positive controls, commercially available Trx1 and (truncated) TrxR1 were used. M: MagicMark XP Western Protein Standard. The fig shows the results of a single representative experiment.(B) Detection of Trx1 in virus particles produced in the presence of the organotellurium compounds: HIV-1 particles which were produced in the presence of 25 μM organotellurium compounds were isolated, lysed and measured for their p24 content. Subsequently, the lysates were measured for their Trx1 content via ELISA using the antibodies MT17R6 and MT13X3-biotin. The values were normalised to the p24 values. The value for the control was then set to 100% in each individual experiment and all other values were adapted against these reference values. The data are means of three independent experiments; the error bars represent the SEM. P-values are indicated if significant (*: p < 0.05; **: p < 0.01; ***: p < 0.001).

HIV-1 obtained from the culture supernatants of HIV-680 1-infected C8166 cells was ultra-centrifuged and the concentrated virus pellet carefully washed and analysed for the presence of Trx1 and TrxR1 by Western Blot with specific antibodies against both proteins. Commercially available positive controls were used to confirm the identity of the detected proteins (Fig 9A). The use of an anti-TrxR1 antibody on viral lysates revealed 3 bands with molecular weights of 55, 60 and 71 kDa. These bands correspond with different isoforms of TrxR1. The TrxR1 control has the expected molecular weight of 55 kDa, corresponding with TrxR1 isoform 2. The band identified as Trx1 (12 kDa) in viral lysates corresponds with the Trx1 control. Although it has been reported that Trx1 is present in the cytoplasm and/or the membrane of a variety of cell types [39–42], this is to our knowledge the first demonstration that both Trx1 and TrxR1 may also be associated with the virus particle.

The viral particles that were produced in the presence of the organotellurium compounds and which showed a reduced infectivity in the TZM-bl assays (Fig 7) were analysed for their Trx1 content by a quantitative ELISA. Firstly, the results (Fig 9B) indicate that Trx1 is present within or on the surface of the virus particle and secondly, the results show that the quantity of Trx1 per unit p24, and thus per virus particle, is invariably reduced by over 50%, when cultivated in the presence of each compound when used at 25 μM. This indicates a possible interference of the compounds with the incorporation of Trx1 into the viral particles during their assembly.

## Discussion

This study was initiated to investigate the role of TrxR1-dependent reduction of gp120 during HIV-1 entry, and the potential of the oxidoreductase system to act as a target for the anti-HIV activity of TrxR1 inhibitors. Previous studies have demonstrated that the TrxR1 inhibitor auranofin inhibits HIV infection through inhibition of gp120 reduction [7]. However, its anti-HIV activity is only evident at concentrations close to its cytotoxic concentration in CEM cell cultures and only approximately 10- to 20-fold lower than its toxicity threshold in PBMC, which results in a moderate selectivity index. Here, the anti-HIV activity of a series of novel organotellurium-containing compounds previously reported as TrxR1 inhibitors [20–22] was studied. It was shown that some of these compounds are active (EC_50_ in the μM range) against HIV-1(III_B_) and HIV-2(ROD) in CEM cells. The four most selective compounds were selected for a more thorough investigation of their mechanism of antiviral activity. These organotellurium compounds differ in their chemical properties such as structure, molecular weight and hydrophobicity/hydrophilicity.

Although the four compounds were found to have a moderate-to-high selectivity index in CEM cell cultures, they are endowed with rather low selectivity indices in PBMCs, using HIV-1(III_B_), HIV-1(BaL) and HIV-1(HE) as infecting virus. A possible explanation for this finding could be the often abundant expression of Trx1 and TrxR1 in tumor cells *versus* primary cell cultures [43]. A certain concentration of TrxR1 inhibitor would only block part of the Trx1/TrxR1-dependent processes in such tumor cells due to the presence of relatively high Trx1/TrxR1 levels. As a result, these type of inhibitors might then be expected to have a lower toxicity in tumor cell cultures. Instead, a similar compound concentration would probably block a much higher amount of Trx1/TrxR1-dependent processes in primary PBMCs that intrinsically may contain lower intracellular Trx1/TrxR1 levels, leading to higher levels of toxicity and thus lower antiviral selectivity indices. Instead, lower Trx1/TrxR1 levels in PBMC cultures may also be expected to result in a higher antiviral activity of the compounds than in the tumor (i.e. CEM) cell cultures, which was not observed in the PBMC culture experiments (Table 2). However, to explain the lower selectivity index in PBMC *versus* CEM cell cultures it should also be kept in mind that the cytotoxicity measurements in PBMC cultures were determined at day 7 post drug exposure, at the time of the read-out of the antiviral effect. In contrast, the toxicity in CEM cell cultures was assessed after 3 to 4 days of cell cultivation. In fact, no cytotoxicity was even observed in 3-day old confluent HeLa cell cultures that were exposed to 100 μM of the test compounds during this incubation period. Thus, the longer the drug-exposure time, the higher drug toxicity becomes evident in the cell cultures.

In order to investigate the mechanism of antiviral action of the organotellurium compounds, their inhibitory effect on different stages of the HIV life-cycle was studied, including entry, reverse transcription, integration, proteolytic cleavage of viral proteins and viral export. A thorough investigation of several possible targets was important since TrxR1 inhibitors may intervene on multiple cellular processes that involve the thioredoxin system such as cell function, cell proliferation, antioxidant defense and redox-regulated signalling cascades [10]. We showed that the herein presented compounds did not have a significant effect on the viral enzymes RT or protease in the *in vitro* (cell culture) experiments. Instead, the inhibition profile of the organotellurium compounds in the HIV-infected TMZ-bl and ACH-2 cell cultures pointed to an antiviral target at a replication event well before proviral DNA incorporation. The time-of-drug-addition studies revealed an inhibition at a very early stage in the viral infection cycle and pointed to the viral entry process as a most likely antiviral target of the compounds. Although viral uncoating (occurring after viral entry but before RT activity) might be influenced as well by oxidoreduction, we assume TE-2 to most likely interfere with a step coinciding with, or resulting in, the fusion of the cellular and viral membranes since TE-2 displayed a profile that was similar to the profile of the fusion inhibitor T-20 (Fig 4A and 4B). These findings are corroborating the observed pronounced interaction with TrxR1 (visualized by SPR) and inhibition of reduction of the disulfide bonds of gp120 by the compounds.

It is somewhat puzzling why the organotellurium compounds were unable to prevent syncytium formation in the HuT-78/HIV-1 + Sup-T1 cocultivation assay whereas compounds that directly interact with the viral envelope or cellular (co)receptors do prevent this event. Also auranofin, a drug that is known to be a TrxR1 inhibitor that inhibits the reduction of disulfide bonds in the viral gp120 [7], could not prevent syncytia formation in this assay system. Despite not having a solid explanation for this differential behaviour of the oxidoreductase inhibitors, the most likely explanation would be that the sensitivity of the two types of assays are different. An indication for this would be that 625 nM of the CXCR4 inhibitor AMD3100 is fully inhibitory in the time-of-addition assay, but for the syncytia formation the IC_50_ is ~ 15-fold higher (9.3 μM). We have also observed such differences (10- to 50-fold) when the inhibitory potential of CBAs such as HHA, GNA or actinohivin have been determined for cell-free virus infection of T-lymphocytes *versus* syncytium formation in cocultures of persistently HIV-1-infected and uninfected cells [44, 45]. Such difference in sensitivity can be due to several reasons. The stoichiometry of syncytia formation is quite different from virus infection since there are many more Env (and CD4/coreceptors) molecules spread across the cell surface compared to the limited number (approximately 10) of Env molecules on a single virion. Also, the biophysics of membrane curvature are strikingly different for cell-cell fusion compared to virus-cell fusion, which may also impact the effects of the drugs.

Although the exact mechanism of action of auranofin in inhibiting virus infection/replication is still unclear, it has been hypothesised that it might inhibit TrxR1 in the recycling (oxidoreduction) system that is required for efficient gp120 reduction during viral entry [7]. When the TE-compounds were investigated for their inhibitory effects on gp120 reduction by the Trx1/TrxR1 oxidoreduction system, they proved equally inhibitory as auranofin. Our SPR studies are in agreement with these findings and revealed that no direct interaction could be detected between the organotellurium compounds and gp120, gp41, soluble CD4 (sCD4) or human serum albumin, whereas a specific dose-dependent interaction with TrxR1 did occur.

Although the organotellurium compound interaction seemed to be quite specific for TrxR1, there was no straight correlation between TrxR1 binding efficiency and eventual antiviral activity of the test compounds. This can be due to the rather simplified assay conditions (i.e. lack of co-substrates such as NADPH). Alternatively, since the organotellurium compounds were quite different in nature, it cannot be excluded that the degree of TrxR1 inactivating activity by the compounds depends on the molecular microsite where they interact with the reductase enzyme. Also, the additional contribution of other cellular oxidoreductase systems in the conformational changes of gp120 such as Grx1/GR or PDI can also play a role in the lack of a straight correlation between TrxR1 inhibition and anti-HIV activity. In fact, we could indeed demonstrate a specific dose-dependent interaction of TE-2 with PDI which proved comparable to the interaction shown with TrxR1.

It is interesting to notice that Trx1 could be detected by ELISA in the virus particles. Additional Western Blot analysis on HIV particles support these findings; however the Western Blot experiments could not unambiguously exclude cell-derived contamination to contribute to protein detected in the virus particles. In fact, although the cell membrane- and microvesicle-derived CD45 could not be demonstrated to be present in the virus lysates, traces of cytosolic markers such as tubulin and GAPDH could still be detected in the virus lysates, and thus, a cytosolic contamination of Trx1 and TrxR1 in the Western Blot analyses could not be excluded. Ideally, an alternative method such as immunogold electronmicroscopy should be performed to support our findings for which Western Blot and ELISA detection of Trx1 and TrxR1 were used, and this will be a focus of future investigations. Although the currently used virus purification methodology did not allow to exactly localize the enzymes in the virus particles, the detected Trx1 is most likely located in or near the viral envelope, where the reduction of gp120 has to occur during the entry/fusion process. In fact, it has previously been reported that human Grx1, another protein that is known to be involved in oxidoreduction of cellular proteins where disulfides are involved, is localised inside the HIV particle as well as at the surface of the virion [46]. Grx1 was suggested to be implicated in the regulation/maintenance of protease activity in the HIV-infected cells. The likely association of Trx1 with the virus particles may therefore be suggestive for an instrumental role in the disulfide oxidoreduction of gp120 during the virus entry process. This assumption is also in agreement with our findings that virus released from drug-treated persistently HIV-infected cell cultures is less infectious. This decreased infectivity could be due to a decrease in Trx1 content of drug-exposed viral particles and/or by the presence of the drug, bound to Trx1/TrxR1 in the budding virus particles. In this respect, the lower infectivity potential of HIV-1 was particularly striking for TE-14-exposed virus cultures, possibly due to the pronounced hydrophobic nature of this compound, and thus its potential higher tendency to adhere to other hydrophobic entities in the viral particle. The phenomenon that an antiviral drug is retained by the budding virus particle in drug-exposed cell cultures has been observed before for the HIV-1 reverse transcriptase inhibitor UC-781, also resulting in the release of less infectious virus particles [47]. However, other techniques should be applied to unambiguously demonstrate retained drug in the virus particle, the exact localisation and function of Trx1/TrxR1 in the virus particles if indeed present or whether Trx1/TrxR1 is closely associated to the viral envelope.

In contrast with most currently available drugs, the organotellurium compounds seem to exert their antiviral efficacy by targeting a functional cellular factor, being redox-regulating enzyme(s) that affect the oxidoreduction of gp120. This action is necessary for efficient entry of the HIV particle into its target cells. Although targeting a cellular function is not a guarantee of delayed or lack-of drug resistance development, a specific inhibition of redox-regulating enzymes to block the required conformational changes in gp120 during the entry process might be attractive in terms of potential viral drug resistance development. In fact, the disulfide/thiol oxidoreduction of gp120 is strictly required during virus entry. Gp120 contains 9 disulfide bridges of which at least 7 are absolutely required for well-functioning of HIV. Site-directed mutagenesis, separately deleting each of the individual disulfide bridges by mutating the involved cysteine, results almost always in mutant non-infectious virus particles [48]. Thus, it seems that the virus may not be able to survive without an intact set of disulfide bridges. Therefore, escaping or circumventing the inhibitory action of drugs targeting redox-regulating enzymes that affect the conformational changes of gp120 is unlikely to occur easily. Long-term drug exposure experiments may address this issue.

In summary, organotellurium compounds have been identified as novel HIV entry inhibitors that act at a very early stage of viral entry, presumably inhibiting the reduction of HIV gp120 through interaction with cellular oxidoreductases such as TrxR1. The TrxR1/Trx1 system may be considered as a potential new target for anti-HIV compounds such as organotellurium derivatives since this cellular function proved necessary for efficient entry of the virus into its target cells.
